# A case of primary testicular lymphoma with continuous spread along the gonadal vein and spermatic cord

**DOI:** 10.1259/bjrcr.20180063

**Published:** 2018-09-08

**Authors:** Kivraj Singh Sabarwal, Ehab Hanfy Mahmoud Ismail

**Affiliations:** 1 Clinical Radiology Registrar, Surrey and Sussex Healthcare NHS Foundation Trust, Surrey, , UK; 2 Consultant Radiologist, Surrey and Sussex Healthcare NHS Foundation Trust, Surrey, , UK

## Abstract

Primary testicular lymphoma (PTL) is a rare form of non-Hodgkin’s lymphoma more prevalent in males aged over 60 years old. PTL has a tendency to disseminate to systemic extranodal sites, however there has been a rare continuous spread involving the gonadal vein and spermatic cord. This method of dissemination has been described in 3 previous cases, and this case report presents another such case where such spread was noted, in a patient with a previous history of seminoma. Knowledge of this method of spread may increase the index of suspicion of PTL on cross-sectional imaging.

## Introduction

Primary testicular lymphoma (PTL) is a rare form of non-Hodgkin’s lymphoma accounting for 5–9% of all testicular cancers.^1,2^ It is most commonly diagnosed in males aged over 60, and the most common finding on histopathological analysis is a diffuse large B-cell non-Hodgkin’s lymphoma.^1,2^ The method of spread of PTL has been suggested to be via both the haematogenous and lymphatic route,^3^ with a tendency to disseminate to systemic extranodal sites, for instance the contralateral testis, central nervous system, lungs, pleura and soft tissue.^4^ There has however been a rare phenomenon associated with PTL, whereby continuous spread along the spermatic cord and gonadal vein has been reported in three cases.^5,6^

With this rare method of spread of PTL in mind, we present a case of a patient with a background of previous testicular seminoma who was referred to our hospital with a left sided scrotal swelling; which was later found to be a PTL with spread up the left gonadal vessels.

## Clinical presentation

A 70-year-old male was referred from primary care to our urology team with a new left sided scrotal lump. This was noted approximately 6 weeks prior to his presentation; and although not particularly painful, had increased in size. His background history, aside from hypertension and eczema, included a history of a right sided orchidectomy and chemotherapy for a testicular seminoma approximately 25 years ago.

On examination he had a large left hemi-scrotal swelling, with the left testis being impalpable.

## Investigations

A baseline set of blood tests which showed a haemoglobin of 131 g l^−1^, normal inflammatory markers, lactate dehydrogenase (LDH) of 743 iu l^−1^ (raised), alpha-fetoprotein (AFP) of 4 ku/L (normal) and a beta human chorionic growth hormone (β-hCG) of 3 iu l^−1^ (upper limit of normal 2 iu l^−1^).

**Figure 1.**
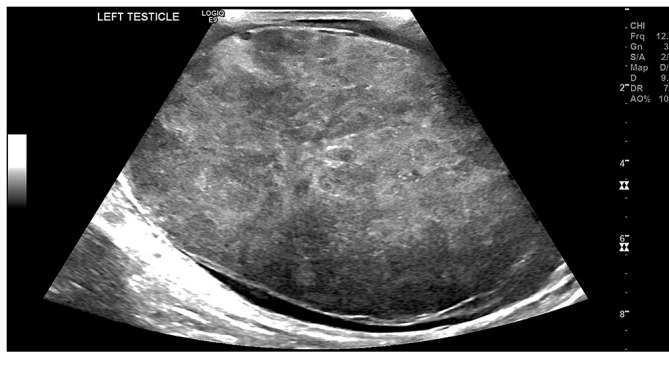
B-Mode ultrasound image of the left testicle which shows a diffuse, heterogenous echotexture.

**Figure 2.**
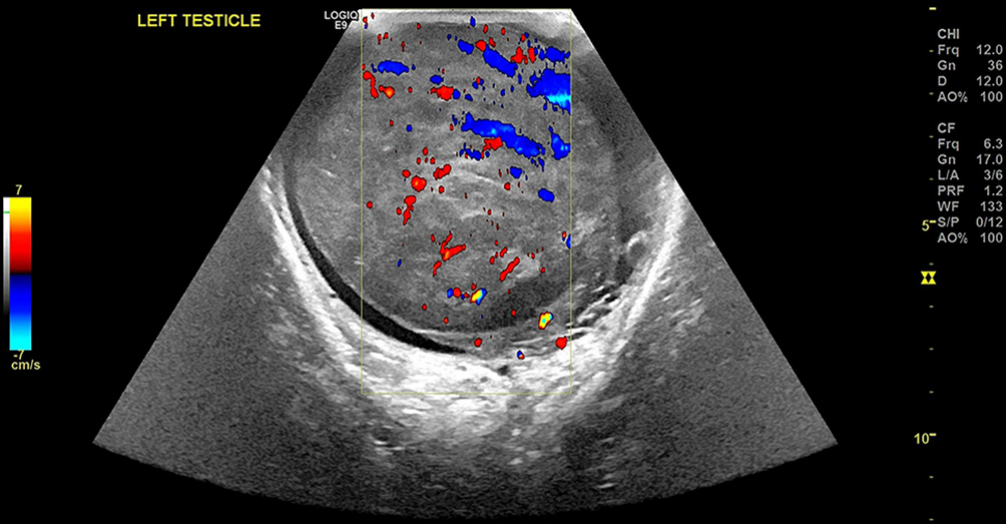
Colour doppler with the spectral gate over the left testicle demonstrating disorganised hypervascularity.

An ultrasound scan of his testis was requested initially, which demonstrated a grossly enlarged testicle with a heterogenous sonographic appearance. The normal testicular architecture was not perceptible [Figure 1], and colour doppler demonstrated a chaotic intra testicular perfusion pattern [Figure 2].

**Figure 3.**
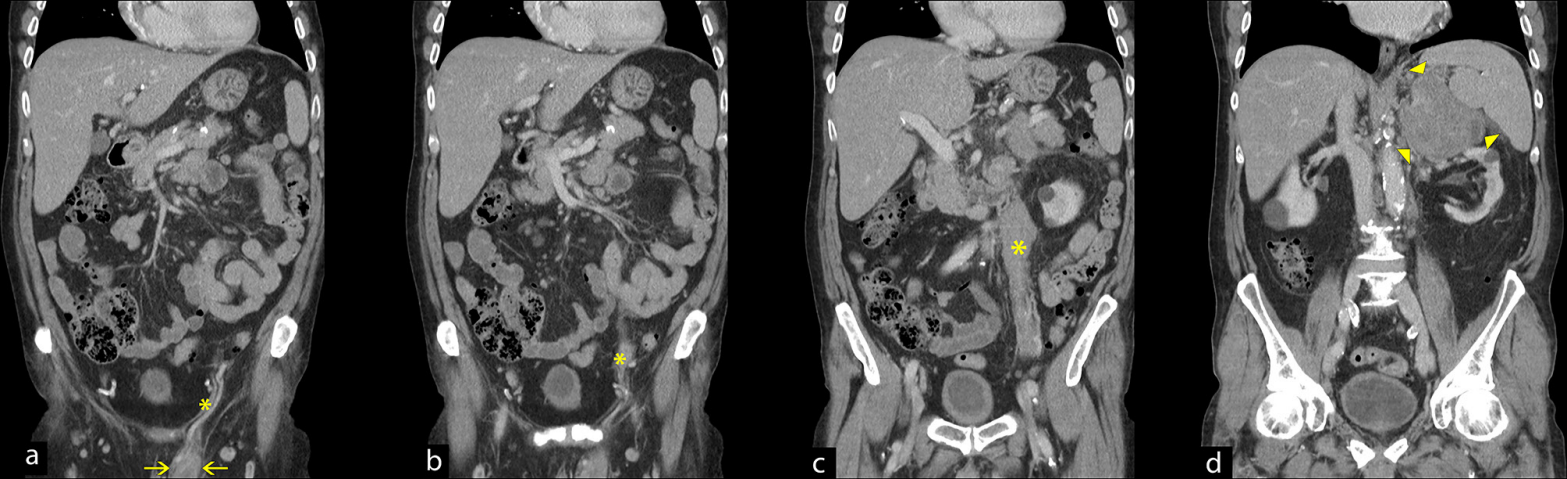
Contrast-enhanced coronal reformats of the abdomen and pelvis taken sequentially (anterior to posterior from the left). The initial image (a) demonstrates the left inguinal mass (arrows) which is seen to extend through the spermatic cord and infiltrates the left gonadal vein (a-c) (asterisk). A further mass is seen above the left kidney (d) (arrowheads).

Following a multidisciplinary team discussion, a contrast enhanced staging CT of his thorax, abdomen and pelvis was performed. This CT demonstrated a soft tissue mass within the left testicle extending through the left spermatic cord and forming a continuous soft tissue column, most likely within the left gonadal vein. This extended to the level of the left kidney, and a further large soft tissue suprarenal mass was noted [Figure 3].

**Figure 4.**
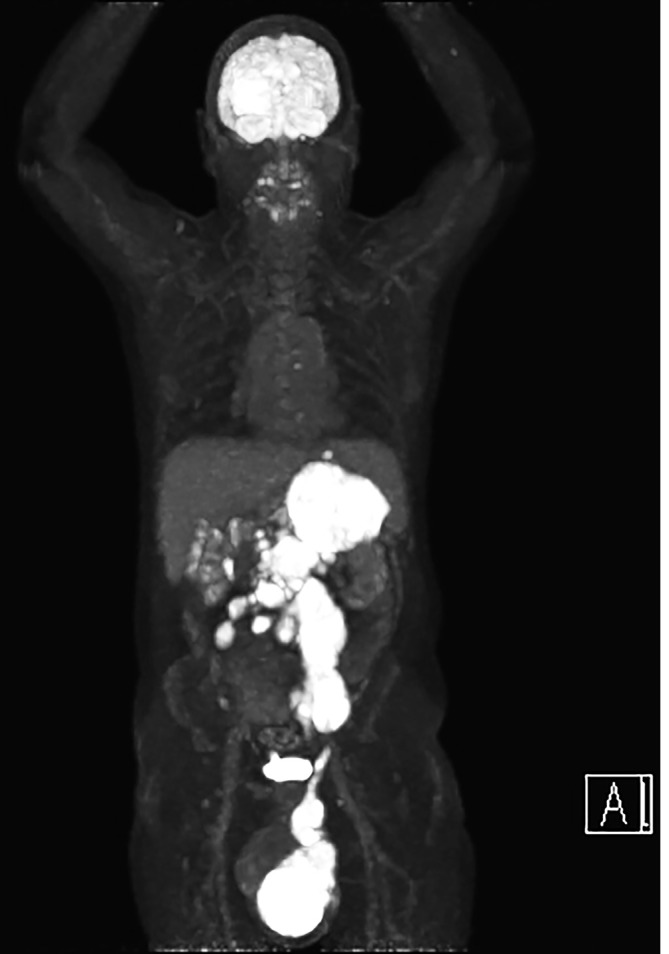
3D reconstruction following the FDG-PET scan which demonstrates the extent of the tumour spread.

**Figure 5.**
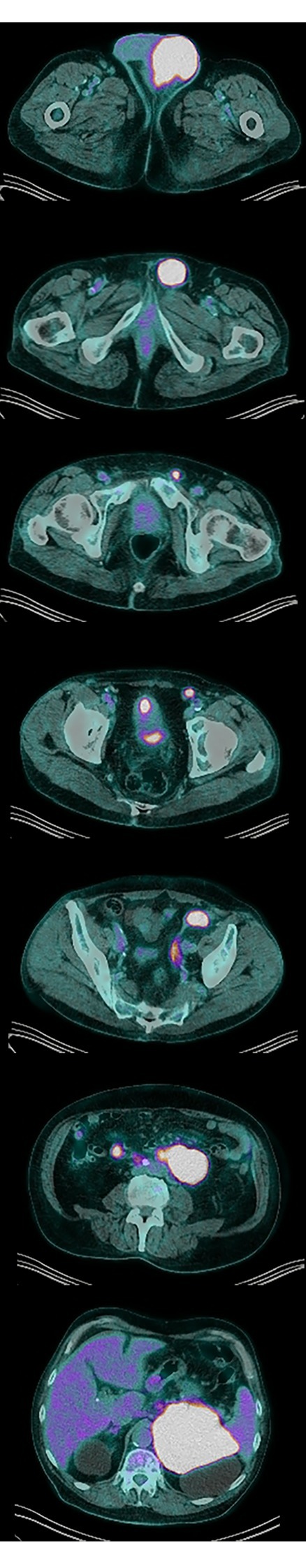
Serial axial slices from the FDG-PET scan demonstrating high uptake by the tumour and infiltrated structures.

After further discussion, a biopsy to obtain a tissue sample and a fluorodeoxyglucose positron emission tomography (FDG-PET) scan for further staging were both arranged. The FDG-PET scan demonstrated the left testicular mass which was FDG avid. The soft tissue noted extending from the testicular mass was also FDG avid along with the left suprarenal mass. [Figures 4 and 5].

Initial CT guided biopsy from the left suprarenal mass was arranged, however, it was felt at the time that it was inaccessible. Therefore, after discussion with the referring team, an ultrasound guided core biopsy from the left testicular mass was performed instead. The tissue cores were found to show features highly suspicious of a high grade diffuse large B cell Non-Hodgkin’s lymphoma of non-follicle centre type (ABC).

## Treatment

Following the histological analysis from the biopsy, our patient proceeded to have a radical left orchidectomy. The histopathological analysis confirmed the presence of the lymphoma as described above, with invasion of the tunica albuginea, tunica vaginalis, epididymis, spermatic cord and the vascular space.

He has since been referred to our haematology team for further treatment.

## Differential diagnosis

This particular case presented interesting diagnostic challenges. Perhaps the biggest confounding factor was out patient’s history of previous right seminoma operated on 25 years ago.

Given our patient’s clinical presentation, following the initial ultrasound, the possibility of seminoma recurrence could be considered. However, the risk of this occurring is 2–5% in the first 15 years of treatment and higher in patients under the age of 30.^1^ Thus, in our patient the probability of recurrence was unlikely. The other differentials given the ultrasound findings alone would be of other non-seminomatous germ cell testicular malignancies; but given our patient’s age these would be unlikely.^1^

Once the cross-sectional imaging had been performed, it was evident that our patient had more advanced disease. This would mean distinguishing PTL from a lymphoma arising from another site with secondary testicular involvement; which would be difficult based on imaging alone.^2^ However, given the nature of the spread: contiguous along the left gonadal vein^3,4^ and the clinical presentation of our patient (painless testicular swelling in the absence of B-symptoms), PTL was the likely diagnosis.

## Discussion

Primary testicular non-Hodgkin’s lymphoma is a rare form of testicular cancer accounting for approximately 5% of all testicular cancers.^5,6^ The presentation is usually a unilateral mass within the testicle, and the presence of constitutional symptoms at diagnosis are uncommon; but may indicate the presence of systemic disease if present.^6^ The main risk factor for developing PTL is in patients infected with the human immunodeficiency virus (HIV), where the incidence is increased and the age of occurrence is often younger.^7^

In addition to diffuse large B-cell lymphoma, there are other subtypes which may be found on histological analysis including Burkitt and follicular lymphoma^5–7^

With regards to initial radiological appearances, sonography of the affected testis may appear as a diffusely hypoechoic but hyperaemic testicle or a focal testicular mass with increased blood flow.^3,6^ The closest differential would be of testicular seminoma; however, the important discriminator is patient age.^3,5,6^ Following the initial sonography, a CT scan would be indicated for staging and to aid further work-up. The finding of continuous infiltration of the gonadal vein is in keeping with PTL given its propensity for haematogenous spread^2^ and has only been described in 3 cases to our knowledge.^3,4^ If an FDG-PET study is carried out; the main findings are usually asymmetric, focal, increased uptake in the affected testicle; and also, may include abnormal increased uptake within inguinal and retroperitoneal lymph nodes particularly at the renal hilum.^8^ It is worth mentioning that physiological FDG accumulation within the testes can vary significantly and can be asymmetric hence mimic disease.^8^ With our patient this was not an issue given his previous history of orchidectomy.

The use of MRI has also been shown to further characterise testicular masses particularly where initial ultrasound evaluation is inconclusive about the classification of a lesion; with one study reporting a sensitivity of 100% and specificity of 97% in classifying malignant lesions.^9^ Specific to PTL, the findings on ultrasound would usually suggest the diagnosis,^10^ with the homogenous mass lesion replacing the affected testis manifesting on MRI as hypo-intense on *T_1_* weighted sequences and iso- to hypo- intense on *T_2_* weighted sequences relative to the unaffected testis.^3,10^ In addition, moderate to strong diffusion restriction may be seen^3^ and low-level enhancement relative to the normal testis following contrast administration.^10^ Due to the aggressive nature of PTL, extension out of the testis and involvement of the epididymis may be seen on MRI.^5,10^ With our patient, MRI was not performed in part due to local protocols, and also following discussion at the urology MDT, the decision was made to obtain a histological sample given the imaging findings on ultrasound and CT to help guide further management.

Staging the disease is mostly based on the Ann Arbor system, with particular analysis of the CNS (including CSF analysis to detect occult disease) and the contralateral testis.^5–7^ Serum biochemical markers particularly the LDH is useful to gauge how aggressive the tumour is, and later, to monitor the response to therapy.^7^

The treatment usually consists of performing and orchidectomy initially which would be a therapeutic intervention (to relieve the tumour burden) and also diagnostic (for histological analysis to confirm the nature of the disease).^11^ This would be followed by either chemotherapy, radiotherapy or a combination of the two,^2,11^ with the precise regimen based on the stage of the disease.^7^ Unfortunately, PTL are very aggressive malignancies, and most patients with Stage I/II disease will experience relapse.^5^ Most relapses occur within the first 2 years of follow-up, but late relapses have been also reported mainly affecting the CNS and the contralateral testis, the former being more common.^6,11^ This has raised the need for CNS prophylaxis to prevent recurrence, however the method of delivery (intrathecal or systemic) remains controversial.^6,7^

## Learning points

1. In older patients presenting with unilateral testicular enlargement with no constitutional symptoms (fever, weight-loss, night sweats), PTL is to be considered in the differential diagnosis.
2. Spread of a testicular tumour through the spermatic cord and the gonadal veins is considered pathognomonic for Primary Testicular B-cell Lymphoma.
3. Initial ultrasound imaging of a scrotal lump may suggest PTL as a differential, however if there is still uncertainty regarding the nature of a testicular lesion, MR imaging may be considered for further accurate characterisation.
4. Unfortunately, due to the aggressive nature of PTL, there is a high propensity of relapse which would most commonly affect the CNS.
